# Supplementary material for: Time to death and its predictors among under-five children on antiretroviral treatment in public hospitals of Addis Ababa, Addis Ababa, Ethiopia, a retrospective follow up study
Source: PLoS One. 2023 Jul 20;18(7):e0288475. doi: 10.1371/journal.pone.0288475 (PMC10358914; doi:10.1371/journal.pone.0288475)
Supplement: Attachment — Submitted filename: Comments.docx [file pone.0288475.s002.docx]

**Comments**

**Abstract**

- The conclusion section should be revised. As it stands now, it is a rehash of the results rather than a conclusion. What is your recommendation? The conclusion must be drawn towards showing the implications for policy and practice

**Introduction**

- Antiretroviral therapy for HIV-infected children leads to immunological reconstitution, as demonstrated by an increase in CD4 cell counts, a lower risk of opportunistic infection, and a longer survival time. Cite it

**Method section**

- The sample size was calculated using statistical STATA Version 14 software, the Sample size was determined by using STATA version 14 statistical software, these ideas seem repetitive
- The maximum sample size from calculated predictors was used. What were these predictors? Where did you get the predictors? You should have to provide the full evidence
- All research participants' records were selected based on the eligibility criteria. Clearly define these eligibility criteria
- “I am not clear with the definition you have placed for the Advanced WHO clinical stage.

**Result**

- More than half 271(65.3%) had good ART adherence, and 333(80.2%) HIV infected under five children born from mothers who had PMTCT service initiated during their period of pregnancy. Split these sentences
- In Table 2, replace Baseline clinical with “Baseline clinical characteristics”
- Replace see Fig 6 with **Fig 6**
- Children who had not used Cotrimoxazole had a higher probability of life as compared to those who had used it for the first two months. But they had a lower probability onwards starting from third months to 34 months better to replace with had a higher probability of survival
- For the selected variables, their collinearity was checked and there was no multi-collinearity among these independent variables. How you checked? What was the result? I think it is better to incorporate them into the document
- As per the finding from the multivariable model, severely underweight children were the Hazard of death 3.19 times more likely to die as compared to normal children (AHR =3.19; 95%CI: 1.32–7.71), additional Children who had tuberculosis were Hazard of death at 3.862 times more likely to die as compared to those children who had no tuberculosis (AHR = 3.86; CI: 1.76– 8.47). Remove unnecessary conjunctions ……I think it is better to rewrite in a simpler and clear way m and put different ideas in separate sentences.

**Discussion**

- The discussion does not explain why factors contribute to time to death. You should have to elaborately discuss each of your findings in this section.
- The strength and limitations of the study should be stated

**General comments**

- The manuscript would benefit from proofreading for grammatical and typographical errors.
