# Supplementary material for: Time to death and its predictors among under-five children on antiretroviral treatment in public hospitals of Addis Ababa, Addis Ababa, Ethiopia, a retrospective follow up study
Source: PLoS One. 2023 Jul 20;18(7):e0288475. doi: 10.1371/journal.pone.0288475 (PMC10358914; doi:10.1371/journal.pone.0288475)
Supplement: Attachment — Submitted filename: Response to Reviewers.docx [file pone.0288475.s003.docx]

**Date March 11, 2023**

**Manuscript ID:** PONE-D-22-31887

**“Time to death and its predictors among under-five children on antiretroviral treatment in public hospitals of Addis Ababa, Ethiopia, A Retrospective Cohort Study."**

**Dear Editors and reviewers**

Thank you for sending us your valuable comments, which immensely improved our manuscript. We included all the editorial comments raised and we also enclosed the point-by-point response of attached here with. It is my pleasure to inform you that the manuscript was edited meticulously.

Respectfully,

Dawit Misganaw

Corresponding author

**Dear Editors:**

- A clean, revised main document was submitted.
- Corrected text within the document was a highlighted in the revised main document.
- The paper revised and edited by a person proficient in written English edits the manuscript.

HIV is an infection caused by the Human Immunodeficiency Virus (HIV), which primarily targets the immune system of a person. HIV infected under five children has rapid disease progression and opportunistic infections due to immune weakness, that goes to higher morbidity and mortality. If not treated, up to 30% of children will die before their first birthday and up to 50% will die before their second birth day.

Worldwide under-five children death due to HIV/AIDS accounted for 1.4% in 2015 and over 120,000 children died due to AIDS-related illnesses in 2016, this equates to 328 deaths every day. In fact, children aged 0–4 years living with HIV are more likely to die than any people living with HIV of any other age. Despite this significant progress, the number of under-five children becoming newly infected and dead with HIV remains unacceptably high, for instance about 150,000 children became infected with HIV in 2015, down from 490,000 in 2000.

In Ethiopia estimated 722,248 people are living with HIV, current prevalence is 1.16%, with children accounting for around 10%. In 2018 PMTCT coverage, which is assumed to be major preventive factor for under-five child deaths, was still in between 63% to 93%, which needs high effort. Coverage of early infant diagnosis of HIV is estimated at around seventy percent and in 2019 from all causes of under-five mortality HIV/AIDS accounts for 2%.

Although visible efforts aimed at improving HIV infected child survival have driven large reductions in mortality levels among children under- five years of age, presence of persistent and intolerably high numbers of child and young adolescent deaths mean more work remains to be done to address the specific survival needs of children and young adolescents in Ethiopia. So, this study is aimed to determine time to death and its predictors among under-five children on antiretroviral treatment in public hospitals of Addis Ababa, Ethiopia. From 2017 to 2022.

Dear reviewer, there has been a major revision of this manuscript (Abstract, introduction, methods, results discussion and conclusions). The language has been extensively examined to correct grammatical and spelling inconsistencies and the whole structure of the manuscript has been revised. We hope now the manuscript is clear and more acceptable than its previous version. We have tried to present the paper in proper manner according to your comment what to supposed to do so. For this, here we have given our responses to each of the concerns you raised, highlighted by yellow color. Again, we would like to remind our strongest gratitude for your effort for the improvement of this manuscript and all the points were addressed below.

Dear Editor, we tried to resubmit a revised manuscript that addresses the points raised during the review process. We include the following items in the revised manuscript as per your comments:

- A rebuttal letter that responds to each point raised by the academic editor and reviewer(s). We uploaded this letter as a separate file labeled 'Response to Reviewers'.
- A marked-up copy of the manuscript that highlights changes made to the original version. We uploaded this as a separate file labeled 'Revised Manuscript with Track Changes' colored in yellow.
- An unmarked version of revised paper without tracked changes. We uploaded this as a separate file labeled 'Manuscript'.
- Regarding financial disclosure, we included updated statement in the cover letter.
- We upload all figure files to the Preflight Analysis and Conversion Engine (PACE) digital diagnostic tool and we are ensured that all figures were meet PLOS requirements.

**We have also addressed these additional journal requirements**:

1. We ensured that the manuscript meets PLOS ONE's style requirements, including those for file naming.
2. Regarding ethics statement section, the comments are well noted and revision was made.
3. Thank you for the remark, there are no restrictions and the anonymized data set is uploaded as a supporting information file. We would like to mention that anybody who wants the data set can get it from the corresponding author through an email address; otherwise, there are no ethical and legal restrictions. Now as PLOS standard we had uploaded the minimal anonymized data set. You can update the data availability statement.
4. Concerning repository information, we provided for data at acceptance; if the manuscript has accepted for publication, please don’t hold it until we provide the relevant accession numbers or DOIs necessary to access the data. We wish to make changes to Data Availability statement. We described these changes in the cover letter and you can update data availability statement to reflect the information we provide.
5. Thank you for the remark, now ethics statement is written in the methods section of our manuscript only. You can update the ethics statement in the online submission form.

Again, we would like to remind our deepest gratitude for your effort for the improvement of this manuscript.

Kind regards,

Dawit Misganaw; on the behalf of co-authors

## Reviewer Comments to the Authors

**Reviewer #1:**

**Comment:** The manuscript technically sound, and do the data support the conclusion. The analysis was done with appropriate statistical method. They should improve the quality of the English throughout the manuscript, since it needs proofreading for grammatical and typographical errors.

**Response**: thank you for your scholarly comments and suggestions. The comments are well noted and revised as much as we can.

**Abstract:** The conclusion section should be revised. As it stands now, it is a rehash of the results rather than a conclusion. What is your recommendation? The conclusion must be drawn towards showing the implications for policy and practice

**Response**: accepted and revision was made

**Introduction:** Antiretroviral therapy for HIV-infected children leads to immunological reconstitution, as demonstrated by an increase in CD4 cell counts, a lower risk of opportunistic infection, and a longer survival time. Cite it

**Response**: accepted and cited.

**Method section:** The sample size was calculated using statistical STATA Version 14 software, the Sample size was determined by using STATA version 14 statistical software, these ideas seem repetitive

**Response**: accepted and revision was made.

**Method section:** The maximum sample size from calculated predictors was used. What were these predictors? Where did you get the predictors? You should have to provide the full evidence

**Response**: description of sample size calculation was narrated. Now predictors were mentioned and the source these predictors was cited.

**Method section:** All research participants' records were selected based on the eligibility criteria. Clearly define these eligibility criteria

**Response**: accepted and defined

**Method section:** “I am not clear with the definition you have placed for the Advanced WHO clinical stage.

**Response**: Thank you dear reviewer. Correction was made accordingly and WHO staging was used by deleting secondary reference. “Advanced WHO clinical stage: Under-five children in WHO clinical stages III and IV during ART enrollment.”

**Result:** More than half 271(65.3%) had good ART adherence, and 333(80.2%) HIV infected under five children born from mothers who had PMTCT service initiated during their period of pregnancy. Split these sentences

**Response**: accepted and revision was made.

**Result:** In Table 2, replace Baseline clinical with “Baseline clinical characteristics”

**Response**: accepted and corrected.

**Result:** Replace see Fig 6 with **Fig 6**

**Response**: accepted and corrected.

**Result:** Children who had not used Cotrimoxazole had a higher probability of life as compared to those who had used it for the first two months. But they had a lower probability onwards starting from third months to 34 months better to replace with had a higher probability of survival

**Response**: accepted and replaced.

**Result:** For the selected variables, their collinearity was checked and there was no multi-collinearity among these independent variables. How you checked? What was the result? I think it is better to incorporate them into the document

**Response**: thank you dear reviver for the comment. Correction was made and included in the main document. “Collinearity among variables was checked using VIF with a result of less than 4.”

**Result:** As per the finding from the multivariable model, severely underweight children were the Hazard of death 3.19 times more likely to die as compared to normal children (AHR =3.19; 95%CI: 1.32–7.71), additional Children who had tuberculosis were Hazard of death at 3.862 times more likely to die as compared to those children who had no tuberculosis (AHR = 3.86; CI: 1.76– 8.47). Remove unnecessary conjunctions ……I think it is better to rewrite in a simpler and clear way m and put different ideas in separate sentences.

**Response**: Thank you for the highly scholarly comments and revision was made.

**Discussion:** The discussion does not explain why factors contribute to time to death. You should have to elaborately discuss each of your findings in this section.

**Response**: Thank you, the comments are well noted and revised as much as we can.

**Discussion:** The strength and limitations of the study should be stated

**Response**: accepted and revision was made

**General comments:** The manuscript would benefit from proofreading for grammatical and typographical errors.

**Response**: We really thank you for your scholarly comments and suggestions as well as for giving us. The comments are well noted and revised as much as we can.

Kind Regards,

Dawit Misganaw on the behalf of co-authors

**Reviewer #2:**

Comment 1: The manuscript is within the scope of the journal. However, although the paper is not novel, the information generated may help program planners. By using the proper statistical notation, the author might enhance how the data is presented. For ease of interpretation, the author could decide to convert the data into figures. The author might also need to make more edits to the content to improve the language and other terminologies.

**Response:** Thank you very much and overall revision of the document was made.

Comment2: The authors did a fantastic job of putting the manuscript together, but it was tough for me to read line by line because they didn't number the pages or the sentences. This is typical manuscript procedure.

**Response**: accepted and revision was made and line number was provided.
