# Supplementary material for: Time to death and its predictors among under-five children on antiretroviral treatment in public hospitals of Addis Ababa, Addis Ababa, Ethiopia, a retrospective follow up study
Source: PLoS One. 2023 Jul 20;18(7):e0288475. doi: 10.1371/journal.pone.0288475 (PMC10358914; doi:10.1371/journal.pone.0288475)
Supplement: Attachment — Submitted filename: Reviewer Comments_1.docx [file pone.0288475.s004.docx]

**PLOS ONE**

**PONE-D-22-31887R1**

**Manuscript:** Time to death and its predictors among under-five children on antiretroviral treatment in public hospitals of Addis Ababa, Addis Ababa, Ethiopia, A Retrospective Cohort Study

**Comments to the editor and authors**

The results of this study are not novel, but they add to the body of knowledge about the Time to Death and its Predictors among Children Under Five and provide local evidence. The study is quite straightforward and uncomplicated, but there are a number of ways that the content might be improved to be more appropriate for Plos One Journal readers. The language could require some editing and refinement.

Please find the following feedback and advice for your consideration in order to further improve your manuscript:

**Title**

1. Dear author; Regarding the problem identified, there is plenty on knowledge among the scientific community that could be used for clinical and public health decisions. Therefore, **what makes this study unique from other similar studies?**

**Abstract**

2. How did you determine the sample size and analysis the descriptive statistics?
3. Line 33-35, ……. Variables with p-value < 0.25 in bivariable cox-regression analysis was candidate for multivariable cox-regression analysis…. please revise the sentence…. How the candidate variables were plugin into multivariable cox regression…...?
4. Line 47-48, children …...should receive special attention…. what special attention the author has recommended?

**Methods**

5. Line 139-140, Please be consistent with the use of terminology…. Retrospective follow up study or retrospective cohort study... You need to be consistent throughout your document.
6. Line 142-145, It could be preferable if you combined the two sentences into one.
7. Line 160-161, how proportional allocation was done? Need clarification
8. Line 162-163. Finally, each participant was selected using a simple random sampling technique (lottery method) … Could you explain please, how the authors have managed lottery method select study participants?
9. How did you handle confounding factors and interaction terms in your analysis?
10. Could you explain why and how Collinearity among variables was checked? For which variables did you check?

**Results**

11. Line 249, The response rate for this study was 97.4%, as the authors noted. This high of a response rate makes a retrospective kind of study unfeasible. How could this figure be reliable?
12. You may consider to state the variables which were not statistically significant in the model.
13. The recommendations are too simplistic and one would wonder if these are not known already, did the scientific community need your research to come up with these recommendations? The author should reflect on the findings again to deduce recommendations that would influence programme and policy decision-making.
