# Supplementary material for: Time to death and its predictors among under-five children on antiretroviral treatment in public hospitals of Addis Ababa, Addis Ababa, Ethiopia, a retrospective follow up study
Source: PLoS One. 2023 Jul 20;18(7):e0288475. doi: 10.1371/journal.pone.0288475 (PMC10358914; doi:10.1371/journal.pone.0288475)
Supplement: Attachment — Submitted filename: Response to Reviewers.docx [file pone.0288475.s005.docx]

**Date: June 08, 2023**

**Manuscript ID:** PONE-D-22-31887R2

**“Time to death and its predictors among under-five children on antiretroviral treatment in public hospitals of Addis Ababa, Ethiopia, A Retrospective follow up Study."**

**Dear Academic Editor,**

Thank you for sending us your valuable comments, which immensely improved our manuscript. We included all the editorial comments raised and we also enclosed the point-by-point response of attached here with. It is my pleasure to inform you that the manuscript was edited meticulously.

Respectfully,

Dawit Misganaw

Corresponding author

**RESPONSE FOR REVIEWERS/ ACADEMIC EDITOR:**

**General comment**: There were missing, discordant, and incomplete statements throughout the document. As such, it still requires careful language editing before publication.

**Response**: Dear academic editor, the manuscript meticulously revised and edited.

**Specific methodological comments**

**Abstract**

I. The authors should describe the name of the hospitals included in the study

**Response**: Dear, correction was made accordingly and described in the study. Public hospitals in Addis Ababa town administration that provide under-five ART services include Black Lion Hospital, Yekatit 12 hospitals medical college, Saint Petrous Specialized Hospital, Alert Hospital, Tirunesh Beijing general hospital, St. Paulos Hospital, Zewditu Memorial Hospital, and Menelik II Referral Hospital were included in the study. The remaining four hospitals, namely Amanuel Hospital, Eka Kotebe Hospital, Gandhi Memorial Hospital, and Ras-Desta Damtew Memorial Hospital, were excluded because, under-five ART service was not given.

II. The measure of association with its level of precision (aHR with 95% CI)

**Response**: Dear, correction was made accordingly and included in the main documents. See the method section of the abstract section.

III. Line #29-30: good to explain why these statistical computations were performed (Long rank test for comparing survival curves across different baseline variables; do the same for the remaining.)

**Response**: Dear, correction was made accordingly and included in the main documents.

IV. Study period (April 12, 2017, to May 12, 2022)

**Response**: Thank you, the comments are well noted and revision was made.

V. The statistical software’s used for data entry and analysis

**Response**: Correction was made accordingly and considered. Data were entered into EPI data 4.2 and analyzed using STATA 14.

VI. Line # 35: The authors reported that “The study participants were followed for total of 8237 person-hours of risk time”. Why did the authors employ person-hours of risk? Check it. Person-months will be correct based on the result you report in the entire document

**Response**: Thank dear, for the comment. It corrected as the study participants were followed for total of 8237 person-months. This correction considered through the document.

VII. Line #36: the authors reported that “41 (9.9%) of HIV infected children were died”. This value is unclear to me.  Is it cumulative incidence? Or prevalence? Since your data s time to event prevalence is not appropriate estimate. In addition, along with the estimate, you should also report the precision interval.

**Response**: Dear, thank you for pertinent input. Correction was made and highlighted in the main document. This value 41 (9.9%) was the number of deaths (41) among 415 study participants (41/415).

VIII. Since estimating time to death is your main objective, we need to know the median survival time with its precision interval. So, I would recommend if you reported it.

**Response**: Thank you for the recommendation. But in our case the survival curve does not crosses 50% survival. If the curve does not cross 50%, then median survival is undefined. In our case less than half of the individuals have an event; due to this we cannot calculate the median survival time.

**Methods**

I. Line #141: survival status (death or censored) is not appropriate medical. You can state “Patients who had incomplete data on the outcome variables; like Date of treatment initiation….” Were excluded from the study

**Response:** Thank you for the comment. Correction was made and included in the document.

II. Line # 144-151:  The sample size calculation deficit clarification. How the authors compute probability of the event P (E), and number of event (E). I have doubt about the adequacy of the sample size since you are including all the public hospital (8 hospitals)

**Response**: Than you dear for your clarification questions. The study sample size of the study calculated using STATA version 14 statistical software using the formula N = Event(E) /Probability of event (E) by considering the following assumptions; A 95% level of confidence, 5% level of significance, 80% power and 10% assumption of withdrawals. The probability of the event (0.107) and significant predictors of mortality (sex (HR=2.4), advanced WHO stage (HR=5.64), TB co-infection (HR=3.71), and low hemoglobin (HR=4.14)) were used from a previous study done in Metema Hospital of Ethiopia. The maximum sample size (426) was from the first predictor which was sex (hratio(2.4), failprob(0.107), wdprob(0.1)). The maximum size of the study was 426 using sex as a predictor (h ratio (2.4), failprob(0.107), wdprob(0.1)).

III. Line # 156: lottery method is not feasible. Simple random sampling methods can be assured by Lottery method, Computer generated randomization or Random table. For your case computer generated randomization will be feasible using your sampling frame

**Response**: Thank you for constructive comment. Correction was made and highlighted in the main document. A random number was generated based on the identification of each medical chart of children using computer. Then, four hundred twenty-six (426) children's medical records were selected using a computer-generated random sampling technique. Furthermore, replacing incomplete medical charts was taken into account.

IV. Why the authors fail to select some hospitals from the total to make it more feasible

**Response**: Because of ART services was not provided in those hospital (The remaining four hospitals, namely Amanuel Hospital, Eka Kotebe Hospital, Gandhi Memorial Hospital, and Ras-Desta Damtew Memorial Hospital, were excluded because, under-five ART service was not provided in those hospitals).

V. Line #159: “Time to death due to HIV/AIDS after initiation of ART” not appropriate; instead replace it with time to death.

**Response**: Thank for the comment. Correction was made accordingly.

VI. Line #171: the authors should mention the unit of measurement (hour, day, month, year)

**Response:** corrected, unit of measurement mentioned “month” within the document

VII. Line #173: edit the statement by adding “of” next initiation

**Response:** Correction was made accordingly.

VIII. Line #180-181: the authors mentioned that death unrelated to the outcome of interest were consider as censored. First, how do you confirm the death in unrelated the study outcome? Second, even you the cause of death, treating as censored is not appropriate. There are other remedies like computing risk analysis

**Response**: Thank you for pertinent input dear. Correction was made accordingly.

IX. Line #181: lives longer than the study period is not appropriate description (not accepted ethically), you can use “still on follow up”

**Response:** Thank for the constructive input. Correction was made and highlighted in the main document.

X. Line #204: Check the language

**Response:** English language was edited.

XI. The authors should describe who are the data collectors with their qualification

**Response:** Thank you for the comment. Correction was made and included in the document. “Eight BSc nurse data collector and Three MSc nurse supervisors were recruited”.

XII. Line #224: the authors describe “stratified Cox Proportional”, but in the remaining section proportional cox regression model. Should be consistent

**Response:** Correction was made and proportional cox regression model was used consistently within the document.

**Result**

I. Response rate is not appropriate for medical chart review. Use appropriate term. You can say, as per the inclusion criteria, about 97.42% were included for the analysis

**Response:** Correction was made and highlighted in the main document.

II. The authors should mention the reason of exclusion for the 11 medical charts

**Response:** A random number was generated based on the identification of each medical chart of children using a computer. Then after we tried to replace incomplete medical charts three times lastly, we use as non-response.

III. Line #246: “resided” check

**Response:** Comment considered and corrective was taken.

IV. Line #288-289: the authors mentioned that “The Kaplan-Meier curve also showed that as the follow-up time was increased, the survival probability of children was decreased”. Is this the correct interpretation of the figure?  Because the survival function is always decreasing function over time. So please check your interpretation by considering median survival time.

**Response.** Thank dear for the comment. Interpretation of median survival time check and corrective action was taken.

V. line # 291: “41 (9.9 of the total study participants died)” check

**Response:** This part was checked and highlighted in the main document.

**Discussions:**

In the discussion section line 357-415, the author compared the findings with previous studies in terms of similarity or difference. However, the underlying causes for the discrepancies between studies need to be elaborated to increase the depth of the discussion and to allow readers know more about the [situation.in](http://situation.in/) addition, the authors should mention the public health importance of the finding

**Response:** Thank you for the comment. Discussion part was improved and public health importance the finding included in the main at end of discussion. “See the discussion part”
